# Mood Stabilizers for Treating Emotional Dysregulation in Adults with Attention-Deficit/Hyperactivity Disorder (ADHD) with or Without Comorbid Bipolar Spectrum Disorders

**DOI:** 10.3390/brainsci15020182

**Published:** 2025-02-12

**Authors:** Giulio Emilio Brancati, Ugo De Rosa, Anna Magnesa, Francesco De Dominicis, Alessandra Petrucci, Elisa Schiavi, Pierpaolo Medda, Margherita Barbuti, Giulio Perugi

**Affiliations:** 1 Psychiatry Unit 2, Department of Clinical and Experimental Medicine, University Hospital of Pisa, Via Roma 67, 56126 Pisa, Italy; 2 Department of Clinical and Experimental Medicine, University Hospital of Pisa, 56100 Pisa, Italy; 3 Mental Health Centre, Local Health Unit 2, Via San Carlo 2, 06049 Spoleto, Italy; 4 Mental Health Centre, Local Health Unit 2, Viale Trieste 68, 05100 Terni, Italy; 5 Psychiatry Unit 2, Azienda Ospedaliero-Universitaria Pisana, Via Roma 67, 56126 Pisa, Italy

**Keywords:** attention-deficit/hyperactivity disorder, emotional dysregulation, affective instability, bipolar disorder, mood stabilizer, lithium, valproate, lamotrigine, methylphenidate, atomoxetine

## Abstract

**Objectives**: The treatment of emotional dysregulation (ED) poses a major challenge for clinicians managing adult attention-deficit/hyperactivity disorder (ADHD). This naturalistic longitudinal study aimed to evaluate the effects of combining mood stabilizers (MS) with standard pharmacotherapy in this population. **Methods**: Fifty-six adult patients with ADHD, with or without bipolar spectrum disorders, who were followed-up for at least 4 months at Pisa University Hospital were included and grouped based on the prescription of ADHD treatment with prior MS, with conomitant MS and without MS. Changes in self-reported ED, self-reported and informant-reported ADHD severity were assessed using RIPoSt-40, ASRS-v1.1, and CAARS-O:SV. Longitudinal analyses were conducted separately for each group using a pairwise one-sample paired Student’s *t*-test. **Results**: A significant reduction in ED severity was observed in those treated with methylphenidate (MPH) and concomitant MS and in those with atomoxetine (ATX) without MS. Negative emotionality and emotional impulsivity significantly decreased in both these groups, while affective instability only improved in those with MPH and concomitant MS. Self-reported ADHD improvements were significant in all groups receiving MPH, whether with concomitant, prior, or without MS. Significant changes in informant-reported ADHD severity were found in those receiving MPH with concomitant or prior MS. **Conclusions**: The findings highlight the benefits of concomitant MS and MPH treatment on ED, suggest a preferential effect of ATX on negative emotionality, and confirm the effectiveness of MPH for adult ADHD symptoms, regardless of additional treatment with MS. Further studies are needed to explore whether and how MS and MPH may complement each other in reducing ED.

## 1. Introduction

Attention-deficit/hyperactivity disorder (ADHD) is a neurodevelopmental disorder characterized by developmentally inappropriate levels of inattention, hyperactivity, and impulsivity. While mostly diagnosed in children and adolescents, ADHD persistence into adulthood has been increasingly recognized in the last decades [1]. Current diagnostic criteria still place the greatest emphasis on cognitive and behavioral dimensions of the disorder, most evident in the developmental age. However, extreme mood instability, irritability, and emotional reactivity (i.e., emotional dysregulation [ED]) also represent common features of adult ADHD [2,3,4], being present in about 35–70% of patients [5]. 

Importantly, higher ED has been associated with increased psychiatric comorbidity, persistence of ADHD in adulthood and functional impairment [6,7,8]. Notably, deficient emotional self-regulation has been found to predict a lower quality of life and social adjustment [9]. Moreover, emotional lability has been associated with impairments in daily life, independent of inattention and hyperactivity/impulsivity [10], and emotional impulsivity and significantly contributes to general occupational impairment and worse work performance [7,11,12]. In addition, ED has been linked to the development of subsequent bipolar disorder (BD) [13], a relatively specific outcome for a significant proportion of patients with ADHD [14].

Given its prevalence and its relevance in terms of functional consequences, the treatment of ED represents a major issue for clinicians dealing with adult ADHD. The available literature suggests that common pharmacological treatments for ADHD may also be effective in improving ED. Several studies indicated that stimulants, particularly methylphenidate (MPH), may have some efficacy in the treatment of ED in ADHD patients [15,16]. Specifically, some studies demonstrated comparable beneficial effects of stimulants on core ADHD symptoms and ED, also showing positive associations between improvements in ED and other ADHD symptoms [17,18,19,20]. Atomoxetine (ATX) has also shown efficacy in addressing ED in adults with ADHD [21,22,23], although its effectiveness was somewhat lower and less consistent than that of stimulants [16,24].

While equal effectiveness of stimulants in addressing inattention, hyperactivity, impulsivity, and ED align with conceptualizations of ADHD that include emotional symptoms among core psychopathological features [25], a consensus has yet to be reached on how to conceptualize this clinically challenging domain within ADHD [5]. Interestingly, the validity of adult ADHD itself has been recently questioned by some authors due to the significant overlap with affective temperaments, mostly cyclothymia, in a retrospective chart review of patients with mood disorders [26]. 

ED is a common feature of both ADHD and cyclothymia [27] and adults with ADHD who were not diagnosed until adulthood have been found to have higher rates of ED and mood disorders, particularly cyclothymia and BD type 2 when compared to those diagnosed during childhood [28]. These findings are consistent with the risk of misdiagnosis of ADHD in patients with cyclothymia, as suggested by Mauer and colleagues [26]. However, the co-occurrence of ADHD and extreme mood instability may also represent a unique diagnostic entity, distinct from ADHD or cyclothymia alone, for which the optimal treatment strategy has yet to be defined.

In this regard, an increased risk of mood destabilization during stimulant treatment has been reported. In the previously mentioned retrospective study on patients with mood disorders, significant mood improvements were observed in only 2% of patients treated with stimulants (compared to 51% of those not treated with stimulants) and a worsening of mood or anxiety in 27% (compared to 4%). It should be noted that only half of the patients treated with stimulants had been formally diagnosed with ADHD, and, while most received a diagnosis of BD, only half had previously received mood stabilizers (MS) [26]. Indeed, a large register-based study suggested that concurrent treatment with MS prevents a positive association between MPH and treatment-emergent mania in patients with BD [29].

While the use of MS in patients with BD and comorbid ADHD is generally recommended [30], there is also a small amount of evidence that MS may be beneficial for the treatment of ADHD without comorbid BD [31]. Specifically, lithium was found as effective as MPH in addressing learning problems, hyperactivity, and impulsivity, in a small randomized double-blind crossover trial on adults with ADHD [32]. Some efficacy in treating ADHD symptoms and impulsive aggression has also been suggested for carbamazepine and its derivatives [33,34,35], while valproate showed significant efficacy in treating chronic aggressive behavior refractory to stimulant treatment in children with ADHD [36]. Finally, lamotrigine, administered alongside other specific drugs, was found effective on clinical global impression in adults with ADHD and comorbid mood disorders [37].

However, to the best of our knowledge, no studies are available that evaluate whether the concurrent use of MS could help reduce ED in patients treated with common pharmacological treatments for ADHD. The aim of this naturalistic longitudinal study is, therefore, to observe whether a combination treatment with MS is effective in treating ED in adults with ADHD. To do so, we examine changes in self-reported ED symptoms in adult patients with ADHD who were prospectively followed up for at least 4 months after the prescription of treatment for ADHD. Based on previous studies [32,38], we hypothesize a significant improvement in ED in patients concurrently receiving MS with ADHD treatment.

## 2. Materials and Methods

### 2.1. Participants

Participants included adult patients (aged ≥18 years) who were diagnosed with ADHD, prescribed treatment for ADHD, and prospectively followed up for at least 4 months at the outpatient service of the Psychiatry Unit 2, Pisa University Hospital, between January 2018 and April 2024. Subjects were required to have a current diagnosis of ADHD according to DSM-5 criteria. Only patients whose ED severity was assessed at study entrance and at follow-up using the Reactivity, Intensity, Polarity, and Stability questionnaire, 40 items-version (RIPoSt-40) [39], were considered eligible.

The following criteria were considered exclusionary: (1) current major affective episodes according to DSM-5 criteria; (2) intellectual disability according to DSM-5 criteria; (3) having been prescribed specific pharmacological treatment for ADHD for more than 30 days before enrollment; (4) not being eligible for specific pharmacological treatment prescription based on clinical decision. Pharmacological treatment for ADHD or other conditions was prescribed by senior clinicians according to routine clinical practice.

All subjects provided written informed consent for the study participation. The study was carried out in accordance with The Code of Ethics of the World Medical Association (Declaration of Helsinki), and the study protocol was approved by the Ethical Committee of the University of Pisa on 15 March 2018 (N.12712_PERUGI). The sample partially overlapped with that used in a previous study conducted by our group [40].

### 2.2. Assessment

Patients were evaluated by psychiatric trainees with at least two years’ experience in the field of neurodevelopmental disorders, under senior psychiatrist supervision. Educational history, marital status, ADHD presentation, and lifetime psychiatric comorbidity according to DSM-5 criteria were investigated during the first consultation. All psychiatric conditions were diagnosed according to DSM-5, except for cyclothymic disorder, where only criteria A-B and D-G were required. Criterion C was not applied as reported in DSM-5: specifically, while a history of mania was considered exclusionary, a lifetime history of major depressive or hypomanic episodes was allowed, in line with previous observations underlining course heterogeneity in mood disorders [41] and with previous studies from our group [27,28,39].

The Italian version of the Diagnostic Interview for ADHD in Adults (DIVA 2.0), a semi-structured, clinician-administered interview, was used to establish the diagnosis of ADHD [42]. Although the interview was based on DSM-IV criteria, DSM-5 criteria were applied for the diagnosis (i.e., ≥5/9 current criteria for inattention and/or hyperactivity/impulsivity required, and symptoms being present before the age of 12 years).

The RIPoSt-40 self-report questionnaire was used to assess ED [39]. The scale consists of 40 items rated on a Likert scale ranging from 1 (“never”) to 6 (“always”), subdivided into four subscales that assess affective instability, positive emotionality, negative emotionality, and emotional impulsivity. The negative emotional dysregulation (RIPoSt-NED) score, calculated as the sum of the affective instability, negative emotionality, and emotional impulsivity subscales, was used as the main outcome variable. Since no significant differences in RIPoSt-40 positive emotionality scores have been observed between clinical and control samples, this subscale was not included in our analyses.

The Adult ADHD Self-Report Scale (ASRS-v1.1) [43] and Conners’ Adult ADHD Rating Scales—Observer: Screening Version (CAARS-O:SV) [44] were used to assess ADHD symptom severity based on self-report and informant report, respectively, at both baseline and follow-up. The items of the ASRS-v1.1 were rated on a 5-point Likert scale ranging from 0 (“never”) to 4 (“very often”), with a total score range of 0 to 72. The total score of the ASRS-v1.1 and the CAARS-O:SV DSM-IV ADHD Symptoms Total Scale were chosen as secondary outcome measures.

### 2.3. Study Design

Participants were subdivided according to treatment for ADHD (MPH vs. ATX) and with or without MS based on clinical charts. Given that MS have repeatedly demonstrated significant efficacy in managing impulsive behavior and affective dysregulation in previous studies on personality disorders, with effects often becoming evident within 8 weeks of treatment [32,38], participants were divided into three groups according to the following scheme: (a) patients having been prescribed MS for more than 8 weeks prior to enrollment (prior MS); (b) patients being prescribed MS within 8 weeks before enrollment or during follow-up (concomitant MS); (c) patients never being prescribed MS before or during follow-up (without MS). Patients who had been on MS for less than 8 weeks before the end of follow-up were excluded. 

Longitudinal analyses to assess changes in outcome measures were conducted separately for each treatment group. Secondary longitudinal analyses were performed to assess changes in RIPoSt-40 subscales composing RIPoSt-NED to evaluate differential contributions of different ED domains to overall improvement. Additionally, secondary longitudinal analyses on the main outcome measures were performed by combining treatment groups in different ways to achieve greater sample sizes: (1) combining patients treated with prior MS and those without MS to assess the effect of initiating ADHD treatment (vs. initiating ADHD treatment with concomitant MS); (2) combining ATX and MPH or MS groups to assess the overall effect of ADHD treatments independently from MS, and the overall effect of initiating MS independently from ADHD treatment.

### 2.4. Statistical Analyses

All the statistical analyses were performed using R Statistical Software Version 4.3.1 (Foundation for Statistical Computing, Vienna, Austria) in November 2024. Descriptive statistics were used to summarize the demographic and clinical characteristics of the sample. Comparative analyses between treatment groups were conducted using pairwise two-sample, two-sided Student’s *t*-test with false discovery rate correction for continuous variables and Pearson’s chi-squared tests (or Fisher’s exact test) with pairwise Fisher’s exact tests as post hoc analyses for categorical variables. Longitudinal analyses were conducted using pairwise one-sample paired two-sided Student’s *t*-test. Cohen’s d was used as the effect-size measure. A sample size of at least 14 participants is adequate to provide a power of 80% to detect large longitudinal changes (Cohen’s d ≥ 0.8) with α = 0.05. A statistical significance threshold of *p* < 0.05 was set in all the analyses.

## 3. Results

### 3.1. Sample Characteristics

Overall, 59 participants were eligible to participate in the study. One participant, originally treated with MPH, was no longer on treatment for ADHD at the end of follow-up and was excluded from the analyses. Two participants had been on MS, respectively valproate and oxcarbazepine, for less than 8 weeks before the end of follow-up and were excluded as well. 

The final sample consisted of 56 participants (Table 1), comprising 35 males and 21 females aged 18 to 51 years. All participants were of Western European descent, except for two of Eastern European descent, two of South American descent, and one of South Asian descent. Nearly all participants (N = 55, 98.2%) presented with at least one psychiatric comorbidity, with mood disorders being the most prevalent (N = 42, 75%).

Follow-up duration ranged from 19 to 59 weeks, with a median duration of 29 weeks (mean ± SD, 32.71 ± 10.19). At study entry, 30 patients were prescribed MPH, 22 patients ATX, and 4 patients bupropion. During follow-up, the following treatment changes were recorded: four patients switched from ATX to MPH, one patient from MPH to ATX, and, among patients taking bupropion, one switched to MPH and three to ATX. Among the four patients whose treatment changed from ATX to MPH, one changed medication due to poor efficacy and three due to reduced tolerability (i.e., increased anxiety, irritability, nausea). Among the three patients starting with bupropion and switching to ATX, two changes were due to poor efficacy, one to reduced treatment adherence. The only switch from bupropion to MPH was motivated by the occurrence of increased affective lability and irritability. The only switch from MPH to ATX was due to increased anxiety and diaphoresis occurring after 4 weeks of MPH titration up to 60 mg/die. Treatment changes occurred on average after 10.3 ± 8.22 weeks of treatment (median, 9.21 weeks; range, 1.71–25.29 weeks). 

At follow-up assessment, all participants had been on treatment with the same ADHD medication for 30.61 ± 11.08 weeks (median, 28.6 weeks; range, 9.86–59 weeks). Participants who switched to MPH during follow-up (N = 5) had been continuously on treatment with MPH at follow-up assessment for 17.2 ± 7.52 weeks (median, 15.7 weeks; range, 9.86–28.4 weeks). Participants who switched to ATX during follow-up (N = 4) had been continuously on treatment with ATX at follow-up assessment for 22.4 ± 9.44 weeks (median, 20.4 weeks; range, 13.6–35.4 weeks). Accordingly, ADHD treatment groups were subdivided based on medications taken at follow-up: 34 patients were assuming MPH (mean ± SD, 34.71 ± 17.62 mg; median, 30 mg; range, 10–100 mg), 22 patients were assuming ATX (mean ± SD, 53.18 ± 16.65 mg; median, 50 mg; range, 25–80 mg).

Regarding treatment with MS, 12 participants had been prescribed MS for more than 8 weeks prior to enrollment (mean ± SD, 81.04 ± 116.9 weeks; median, 28.6 weeks; range, 8.42–387.71 weeks). This group (prior MS) included five patients on lithium, three on valproate, three on a combination of lithium and valproate, and one on a combination of lithium and oxcarbazepine. At follow-up, six of these patients were treated with MPH for ADHD, while six received ATX. Conversely, 26 participants were prescribed MS concomitantly with ADHD treatment, including 10 patients who received MS on average 3.6 ± 1.9 weeks before enrollment (median, 3.5 weeks; range, 0.86–6.86 weeks), 12 patients who received MS at study entry, and 4 patients who first received MS on average 13.54 ± 8.0 weeks after (median, 10.79 weeks; range, 7.57–25 weeks). This group (concomitant MS) comprised 12 patients on lithium, five on valproate, three on lamotrigine, four on a combination of lithium and valproate, and two on a combination of lithium and lamotrigine. At follow-up, these patients had received MS continuously on average for 28.20 ± 7.68 weeks before enrollment (median, 27.3 weeks; range, 14.43–47.43 weeks); 18 were treated with MPH for ADHD, while 8 received ATX. Finally, 18 participants were never prescribed MS before or during follow-up. At follow-up, 10 of these patients were treated with MPH, while 8 received ATX.

Overall, lithium was the most used MS, with 17 cases alone and 10 cases in combination with other MS, followed by valproate, which was used in eight patients alone and in seven patients in combination with lithium. Treatment with MS (prior or concomitant) was significantly associated with the diagnosis of mood disorders (84.2% vs. 55.6%; Fisher’s exact *p* = 0.044) and bipolar spectrum disorders (78.9% vs. 44.4%; χ^2^ = 5.18, *p* = 0.022). Treatment with MPH vs. ATX was not significantly associated with the MS treatment group (prior MS: 50% with MPH; concomitant MS: 69.2%; without MS: 55.6%; Fisher’s exact *p* = 0.465). No significant differences in the baseline RIPoSt-NED score, ASRS-v1.1 total score, and CAARS-O:SV DSM-IV ADHD Symptoms Total Scale, nor in final MPH or ATX dose emerged among all treatment groups (*p*_adj_ < 0.05).

### 3.2. Main Longitudinal Analyses

Significant improvements in ED and ADHD severity were observed over follow-up in specific treatment groups (Table 2). Particularly, the group of patients treated with MPH and concomitant MS exhibited significant reductions in the RIPoSt-NED score (d = 1.12), ASRS-v1.1 total score (d = 1.12), and CAARS-O:SV DSM-IV ADHD Symptoms Total Scale (d = 1.23). A significant reduction in the RIPoSt-NED score was also found in patients treated with ATX without MS (d = 0.91). As for ADHD severity, the ASRS-v1.1 total score was also significantly reduced in patients treated with MPH with prior MS (d = 1.65) and without MS (d = 1.02), and CAARS-O:SV DSM-IV ADHD, The Symptoms Total Scale also showed significant improvement in those treated with MPH and prior MS (d = 1.40).

### 3.3. Secondary Analyses 

Longitudinal changes in RIPoSt-40 subscales were examined to evaluate which domains of ED contributed to RIPoSt-NED improvements. Patients treated with MPH and concomitant MS demonstrated significant reductions in RIPoSt-40 affective instability (t = 3.53, *p* = 0.003; d = 0.83), negative emotionality (t = 3.67, *p* = 0.002; d = 0.87), and emotional impulsivity (t = 5.04, *p* < 0.001; d = 1.19). Those treated with ATX without MS showed significant improvements in RIPoSt-40 negative emotionality (t = 2.63, *p* = 0.034; d = 0.93), and emotional impulsivity (t = 4.41, *p* = 0.003; d = 1.56), but not in affective instability (t = 0.52, *p* = 0.619; d = −0.18). No other significant changes in RIPoSt-40 subscales were observed.

When the analyses were repeated by combining patients treated with prior MS and those without MS, in order to increase the sample size, a trend to significance was only observed for changes in RIPoSt-NED for both patients treated with MPH (N = 16; t = 1.82, *p* = 0.088; d = 0.46) and ATX (N = 14; t = 2.11, *p* = 0.055; d = 0.56). Conversely, ADHD severity significantly decreased in patients treated with MPH after prior MS or without MS based on the ASRS-v1.1 total score (N = 16; t = 4.96, *p* < 0.001; d = 1.24) and on CAARS-O:SV (N = 13; t = 2.27, *p* = 0.044; d = 0.66). No significant changes were observed, instead, in patients treated with ATX after prior MS or without MS based on ASRS-v1.1 total score (N = 14; t = 1.72, *p* = 0.110; d = 0.46) and on CAARS-O:SV (N = 13; t = 0.69, *p* = 0.502; d = 0.19). 

Improvements associated with ADHD treatments, independent of the MS group, and vice versa, are finally presented in Table 3. 

Significant improvements in RIPoSt-NED were found for all comparisons, with large effects associated with MS initiation (d = 0.86) and with MPH (d = 0.73) and moderate effects in those without or having previously initiated MS (d = 0.46) and with ATX (d = 0.48). ADHD severity based on the ASRS-v1.1 total score was also significantly improved in all comparisons, with comparably large effects in those with and without concomitant MS (respectively, d = 0.94 and d = 0.83). When looking at ADHD treatments, a large effect was associated with MPH (d = 1.19), while a moderate effect was found for ATX (d = 0.52). Improvements in CAARS-O:SV instead mirrored those in RIPoSt-NED, with large effects associated with MS initiation (d = 1.11) and with MPH (d = 0.93) and moderate effects in those without or having previously initiated MS (d = 0.43) and nonsignificant moderate improvements in those with ATX (d = 0.43).

## 4. Discussion

This study highlights the benefits of integrating MS, particularly lithium and its combinations, with standard pharmacotherapy in adults with ADHD to address ED symptoms. 

The positive impact of MS initiation was particularly evident in patients treated with MPH. Specifically, while self-reported symptoms of ADHD were significantly, substantially, and comparably reduced in all groups receiving MPH—whether with concomitant MS, prior MS, or without MS—a significant and pronounced reduction in ED severity was observed only in those treated with MPH and concomitant MS. Delving deeper, only patients receiving MPH and concomitant MS showed significant and large reductions across all facets of ED assessed, namely affective instability, negative emotionality, and emotional impulsivity. Changes in informant-reported ADHD severity, instead, were significant in patients receiving MPH either with concomitant or prior MS but not in those without MS, whereas a significant effect emerged when the groups with prior MS and without MS were combined.

Among participants treated with MPH without MS, a lower baseline ED severity might have partially explained the lack of significant longitudinal changes. However, the RIPoSt-NED score in this group was still approximately 1.5 standard deviations higher than what had been previously observed in a non-clinical sample [39]. Patients who had been on prior MS, instead, showed ED severity levels similar to those who initiated MS, leaving potential room for the effectiveness of additional MPH treatment. In this group, a large reduction in ED severity was found, but it was only associated with a trend toward significance. Although these results were likely influenced by the smaller sample size compared to the group with concomitant MS, no significant improvements in ED severity were noted when the groups with prior MS and without MS were combined to increase power. 

Overall, these results support the effectiveness of MPH for ADHD symptoms, regardless of additional treatment with MS, and provide no evidence of detrimental effects of MPH on ED. Moreover, they highlight the benefits of concomitant MS and MPH treatment on ED and underscore the need for further studies to explore whether and how MS and MPH may complement each other in reducing ED. Specifically, a controlled design allocating patients with similar severity to groups receiving MS followed by MPH, MPH followed by MS, and concomitant MS and MPH would be required to test the hypothesis of the additive effects of these treatments. 

A different pattern of findings was observed in participants treated with ATX. In this smaller group, a significant reduction in ED severity was found among those not receiving MS. Improvements were noted in negative emotionality and emotional impulsivity but not in affective instability, where a non-significant worsening was observed. ATX has shown moderate effects on ED in previous studies, although generally less robust than those observed with stimulants [24]. However, significant benefits on anxious comorbidities have been repeatedly demonstrated and may potentially explain the effect on ED facets observed in our sample [45,46]. Conversely, a worsening of mood symptoms has been previously observed in a minority of patients with comorbid BD treated with ATX [47,48]. The counteracting effects of ATX and MS in patients with BD comorbidity may have prevented the observation of significant effects on ED in patients treated with prior or concomitant MS included in our study.

Notably, no significant changes were found in self-reported ADHD severity in ATX-treated groups, while a trend to significance was only observed for changes in informant-reported ADHD severity in patients receiving ATX and concomitant MS. Even in this case, a greater sample size could be needed to observe significant effects. Indeed, when all patients with ATX were combined, a significant improvement in self-reported ADHD severity emerged. A lower and more delayed efficacy of ATX compared to stimulants has been suggested by previous studies [40,49,50] and could have contributed to our findings on ADHD severity. Particularly, given that previous studies have observed variable temporal patterns of response to ATX, including patients who show gradual improvement over periods extending beyond 6 months, it is possible that a longer follow-up duration would have allowed for the detection of stronger ATX effects. This is especially relevant for patients who switched to ATX during the follow-up period.

In summary, our results emphasized the potential benefit of combining MS with ADHD pharmacotherapy for the management of ED. Two strategies emerged as promising approaches to addressing ED. On the one hand, patients receiving MS and MPH appeared to benefit the most in all ED facets, while on the other, ATX was found to be effective on negative emotionality and emotional impulsivity in a group with lower baseline ED severity who was not prescribed any MS. We previously suggested a preferential indication of MPH for patients showing a higher severity of externalizing symptoms, greater affective instability and a higher rate of bipolar spectrum disorders, while ATX may be preferred in those with predominantly inattentive presentations, less severe ED, and comorbid anxiety and depressive disorders [31,40]. While current findings align with this hypothesis, further studies are needed to better distinguish between MPH and MS’s contribution to improvements in ED and ADHD severity.

From a clinical perspective, the present findings first underscore the importance of a tailored approach to ADHD treatment, particularly for patients with comorbid mood disorders or severe ED. For these individuals, the combination of MS and ADHD medications offers a promising strategy to address the broader spectrum of symptoms. This approach may also help mitigate the risks of treatment-emergent mood destabilization associated with stimulants, as highlighted in a prior study [29], and provide additional benefits. While our study referred to the adult population, our findings might also help inform the treatment of pediatric ADHD. Significant symptoms of ED have been observed in 24–50% of youths with ADHD [5]. However, most of the literature focused on irritability or anger-related behaviors in children, while affective instability and internalizing symptoms received less attention. In addition, the adolescent population has been long overlooked, and only a few inconclusive observational studies are available, for instance, on the effects of stimulants on ED in adolescents [15]. Given the relatively young age of our sample and the common co-occurrence of ADHD and bipolar spectrum disorders during the developmental age [14], our results warrant replication in adolescent samples. 

Several limitations must be considered when interpreting these findings. The observational design and naturalistic setting of the study, while reflective of real-world clinical practice, limit the ability to establish causality. Specifically, the absence of a controlled design introduces significant risks of indication bias, which could affect the validity and reliability of our findings. As a result, our conclusions should be considered preliminary and interpreted with caution until further studies are conducted to confirm these results. For instance, due to the naturalistic design of our study, certain relevant variables, such as inpatient or psychosocial treatments received before or during the follow-up period, may have influenced treatment outcomes. These factors were not accounted for in the current study but should be controlled in future research to strengthen the validity of the findings. Additionally, the reliance on self-reported measures for ED, such as the RIPoSt-40, although validated, introduces potential biases and underscores the need for adding clinician- or informant-reported measures in future research. Moreover, a systematic evaluation of the global functioning of our patients was lacking. Understanding which therapeutic strategy offers the greatest long-term functional improvements is crucial, particularly for adults with ADHD. Therefore, future studies should place greater emphasis on this aspect. Finally, the small sample size, particularly within certain subgroups, may have limited our ability to detect mild and moderate treatment effects, underscoring the need for larger studies to provide more robust evidence. 

## 5. Conclusions

This study provides preliminary evidence for the role of MS in managing ED in adults with ADHD, particularly when used in combination with MPH. The absence of existing studies on the effects of MS on ED in ADHD populations underscores the novelty and importance of these findings. Future studies should aim for larger, controlled trials to confirm and expand these results. While further research is warranted, this clinical approach holds promise for improving outcomes in this challenging patient population.

## Figures and Tables

**Table 1:** Characteristics of the sample at study entry (N = 56). Abbreviations: M = mean; SD = standard deviation.

Demographic Variables	M ± SD/N (%)
Age (years)	27.77 ± 8.76
Gender (male)	35 (62.5)
**Marital status**	
Unmarried	49 (87.5)
Married	4 (7.1)
Divorced	3 (5.4)
**Educational history**	
School years	14.14 ± 3.35
Number of grade repetitions	0.96 ± 1.61
**ADHD presentation**	
Combined	32 (57.1)
Predominantly inattentive	23 (41.1)
Predominantly hyperactive/impulsive	1 (1.8)
**ADHD severity (CAARS-O:SV)**	
DSM-IV Inattentive Symptoms	18.56 ± 5.58
DSM-IV Hyperactive/Impulsive Symptoms	12.43 ± 6.45
DSM-IV ADHD Symptoms Total	30.98 ± 10.16
ADHD Index	23.83 ± 5.87
**Self-reported symptoms**	
ASRS-v1.1 total score	49.21 ± 11.01
RIPoSt-NED score	118.43 ± 28.58
**Family history (first degree)**	
Mood disorders	36 (64.3)
Bipolar disorder	18 (32.1)
Neurodevelopmental disorders	17 (30.4)
Anxiety disorders	13 (23.2)
Substance use disorders	9 (16.1)
Suicide attempts	1 (1.8)
Psychosis	1 (1.8)
**Mood disorders comorbidity (lifetime)**	
Any mood disorder	42 (75.0)
Any bipolar or related disorder	38 (67.9)
Bipolar disorder type 1	3 (5.4)
Bipolar disorder type 2	16 (28.6)
Cyclothymic disorder	19 (33.9)
Major depressive disorder	4 (7.1)
**Anxiety disorders comorbidity (lifetime)**	
Any anxiety disorder	25 (44.6)
Generalized anxiety disorder	9 (16.1)
Panic disorder	16 (28.6)
Social anxiety disorder	5 (8.9)
**Substance use disorders comorbidity (lifetime)**	
Any substance use disorder	24 (42.9)
Cannabis use disorder	21 (37.5)
Alcohol use disorder	14 (25.0)
Cocaine use disorder	10 (17.9)
Amphetamine-type stimulant use disorder	4 (7.1)
Opioid use disorder	2 (3.6)
Benzodiazepine use disorder	3 (5.4)
**Other comorbidity (lifetime)**	
Autism spectrum disorder	11 (19.6)
Tourette syndrome	5 (8.9)
Obsessive–compulsive disorder	5 (8.9)
Anorexia nervosa	1 (1.8)
Binge-eating disorder	4 (7.1)
Borderline personality disorder	8 (14.3)

Abbreviations: CAARS-O:SV, Conners’ Adult ADHD Rating Scales—Observer: Screening Version.

**Table 2:** Longitudinal changes in ED and ADHD severity according to ADHD treatment and MS group. *p* < 0.05 are shown in bold italic, *p* < 0.10 are shown in italic. Abbreviations: ATX = atomoxetine; M = mean; MPH = methylphenidate; SD = standard deviation.

**A. RIPoSt-NED Score**							
**ADHD Treatment**	**MS Group**	**N**	**Baseline** **(M ± SD)**	**Follow-Up** **(M ± SD)**	**t**	***p***	**d**
MPH	Concomitant MS	18	127 ± 29.6	104 ± 36.4	4.77	**<0.001**	1.12
MPH	Prior MS	6	127 ± 29.5	108 ± 32.0	2.14	0.085	0.88
MPH	Without MS	10	110 ± 32.3	98.6 ± 36.1	1.00	0.345	0.32
ATX	Concomitant MS	8	119 ± 28.8	107 ± 15.1	1.25	0.253	0.44
ATX	Prior MS	6	114 ± 20.7	106 ± 22.2	0.99	0.367	0.41
ATX	Without MS	8	105 ± 25.0	97.9 ± 26.9	2.58	**0.037**	0.91
**B. ASRS-v1.1 Total Score**							
**ADHD Treatment**	**MS Group**	**N**	**Baseline** **(M ± SD)**	**Follow-Up** **(M ± SD)**	**t**	***p***	**d**
MPH	Concomitant MS	18	53.3 ± 9.15	40.9 ± 14.0	4.74	**<0.001**	1.12
MPH	Prior MS	6	57.0 ± 10.2	38.3 ± 16.2	4.04	**0.010**	1.65
MPH	Without MS	10	49.2 ± 8.28	36.8 ± 11.8	3.22	**0.011**	1.02
ATX	Concomitant MS	8	41.1 ± 12.1	33.9 ± 8.31	1.67	0.138	0.59
ATX	Prior MS	6	46.7 ± 12.9	43.3 ± 13.4	0.65	0.546	0.26
ATX	Without MS	8	44.1 ± 10.7	38.2 ± 13.1	1.81	0.114	0.64
**C. CAARS-O:SV DSM-IV ADHD Symptoms Total Scale**							
**ADHD Treatment**	**MS Group**	**N**	**Baseline** **(M ± SD)**	**Follow-Up** **(M ± SD)**	**t**	***p***	**d**
MPH	Concomitant MS	14	35.7 ± 9.69	20.5 ± 10.1	4.59	**<0.001**	1.23
MPH	Prior MS	6	35.5 ± 8.69	19.0 ± 11.1	3.42	**0.019**	1.40
MPH	Without MS	7	28.1 ± 8.52	26.1 ± 7.43	0.43	0.686	0.18
ATX	Concomitant MS	7	24.1 ± 9.46	13.3 ± 7.23	2.26	0.065	0.85
ATX	Prior MS	5	28.2 ± 12.9	24.2 ± 12.7	0.12	0.909	0.05
ATX	Without MS	8	28.2 ± 8.56	25.1 ± 12.8	0.74	0.484	0.25

Bold values indicate statistical significance with *p* < 0.05.

**Table 3:** Longitudinal changes in ED and ADHD severity associated with ADHD treatments, independent of the MS group, and vice versa. *p* < 0.05 are shown in bold italic, *p* < 0.10 are shown in italic. Abbreviations: ATX = atomoxetine; M = mean; MPH = methylphenidate; SD = standard deviation.

**A. RIPoSt-NED Score**							
**ADHD Treatment**	**MS Group**	**N**	**Baseline** **(M ± SD)**	**Follow-Up** **(M ± SD)**	**t**	***p***	**d**
All groups	Concomitant MS	26	125 ± 29.1	105 ± 31.1	4.40	**<0.001**	0.86
All groups	Prior MS + Without MS	30	113 ± 27.5	102 ± 29.4	2.50	**0.018**	0.46
MPH	All groups	34	122 ± 30.5	103 ± 34.7	4.24	**<0.001**	0.73
ATX	All groups	22	113 ± 24.9	103 ± 21.3	2.27	**0.034**	0.48
**B. ASRS-v1.1 Total Score**							
**ADHD Treatment**	**MS Group**	**N**	**Baseline** **(M ± SD)**	**Follow-Up** **(M ± SD)**	**t**	***p***	**d**
All groups	Concomitant MS	26	49.6 ± 11.5	38.8 ± 12.8	4.81	**<0.001**	0.94
All groups	Prior MS + Without MS	30	48.9 ± 10.8	38.8 ± 12.9	4.56	**<0.001**	0.83
MPH	All groups	34	52.8 ± 9.21	39.3 ± 13.5	6.93	**<0.001**	1.19
ATX	All groups	22	43.7 ± 11.5	38.0 ± 11.7	2.45	**0.023**	0.52
**C. CAARS-O:SV DSM-IV ADHD Symptoms Total Scale**							
**ADHD Treatment**	**MS Group**	**N**	**Baseline** **(M ± SD)**	**Follow-Up** **(M ± SD)**	**t**	***p***	**d**
All groups	Concomitant MS	21	32.5 ± 10.8	18.1 ± 9.7	5.10	**<0.001**	1.11
All groups	Prior MS + Without MS	26	29.7 ± 9.56	23.8 ± 10.8	2.16	**0.041**	0.43
MPH	All groups	27	33.6 ± 9.56	21.6 ± 9.76	4.74	**<0.001**	0.93
ATX	All groups	20	26.9 ± 9.90	20.8 ± 11.9	1.91	0.072	0.43

Bold values indicate statistical significance with *p* < 0.05.

## Data Availability

The data presented in this study are available from the corresponding author on request due to privacy reasons.

## Abbreviations

The following abbreviations are used in this manuscript:

ADHD	Attention-deficit/hyperactivity disorder
ED	Emotional dysregulation
BD	Bipolar disorder
MPH	Methylphenidate
ATX	Atomoxetine
MS	Mood stabilizers
